# Postoperative complications after esophagectomy for cancer, neoadjuvant chemoradiotherapy compared to neoadjuvant chemotherapy: A single institutional cohort study

**DOI:** 10.1016/j.ctro.2023.100610

**Published:** 2023-03-04

**Authors:** Halla Sif Ólafsdóttir, Emmy Dalqvist, Eva Onjukka, Fredrik Klevebro, Magnus Nilsson, Giovanna Gagliardi, Gabriella Alexandersson von Döbeln

**Affiliations:** a Division of Surgery, Department of Clinical Science, Intervention and Technology, Karolinska Institutet, SE-141 52 Huddinge, Sweden; b Department of Radiotherapy, Karolinska Comprehensive Cancer Center, Karolinska University Hospital, SE-171 64 Solna, Sweden; c Section of Radiotherapy Physics and Engineering, Department of Medical Radiation Physics and Nuclear Medicine, Karolinska Comprehensive Cancer Center, Karolinska University Hospital, SE-171 64 Solna, Sweden; d Department of Oncology-Pathology, Karolinska Institutet, SE-171 64 Solna, Sweden; e Department of Upper Abdominal Diseases, Karolinska Comprehensive Cancer Center, Karolinska University Hospital, SE-141 57 Huddinge, Sweden; f Medical Unit of Head, Neck, Lung and Skin Cancer, Karolinska Comprehensive Cancer Center, Karolinska University Hospital, SE-171 64 Solna, Sweden

**Keywords:** Esophageal neoplasm, Radiotherapy, Adverse effects, Postoperative complications, Esophagectomy

## Abstract

**Background:**

Complications after esophagectomy are common and the possible increase in postoperative complications associated with neoadjuvant chemoradiotherapy is of concern. The aim of our study was to analyze if the addition of radiotherapy to neoadjuvant chemotherapy increases the incidence and severity of postoperative complications, including evaluation of the relation between radiation doses to the heart and lungs and postoperative complications.

**Methods:**

The study was based on an institutional surgical database for esophageal cancer. The study period was October 2008 to March 2020. Patients treated with neoadjuvant chemoradiotherapy were compared to patients treated with neoadjuvant chemotherapy and dose/volume parameters for the lungs and heart considered. The primary outcome was 30-day postoperative complications.

**Results:**

During the study period, 274 patients underwent surgery for esophageal cancer, 93 patients after neoadjuvant chemotherapy and 181 patients after neoadjuvant chemoradiotherapy. The median prescribed radiation dose to the planning target volume was 41.4 Gy, the median of the mean lung dose was 6.2 Gy, and the median of the mean heart dose was 20.3 Gy. The addition of radiotherapy to neoadjuvant chemotherapy did not increase the incidence of postoperative complications. Neither were radiation doses to the lungs and heart associated with postoperative complications. Taxane-based chemotherapy regimens were however associated with an increased incidence of postoperative complications.

**Conclusions:**

In our cohort, the addition of neoadjuvant radiotherapy to chemotherapy was not associated with postoperative complications. However, taxane-based chemotherapy regimens, with or without concomitant radiotherapy, were associated with postoperative complications.

## Highlights

• Postoperative complications after esophagectomy for cancer were common.
• Neoadjuvant radiotherapy was not associated with more postoperative complications.
• Neoadjuvant taxanes were associated with increased postoperative complications.

## 1 Introduction

Preoperative chemo- or chemoradiotherapy and surgery, followed by postoperative immuno- or chemotherapy, is the currently recommended curative treatment for esophageal cancer in Europe [1]. Postoperative complications after esophagectomy are however common [2], [3], [4], [5], [6] and the increased risk of postoperative complications after neoadjuvant treatment in observational studies is of concern [7], [8], [9].

Radiotherapy for esophageal cancer inevitably leads to irradiation of vital organs in the vicinity, i.e., the heart and the lungs. Theoretically, preoperative radiation induced tissue damage to these organs could increase postoperative complications and previous studies indicate that radiation to the lungs increases the incidence of postoperative pulmonary complications [10], [11]. Radiation doses to the heart have however not been associated with increased postoperative complications [11], [12]. Neither did four randomized controlled trials, comparing neoadjuvant chemoradiotherapy (nCRT) and neoadjuvant chemotherapy (nCT) [13], [14], [15], [16], demonstrate an increase of postoperative complications associated with the addition of radiotherapy, though more severe postoperative complications were associated with nCRT in one trial [13].

To further investigate the association between postoperative complications and the addition of radiotherapy to nCT we analyzed real-world data from an institutional database. We also evaluated the relation between radiation doses to the lungs and heart and postoperative complications as radiation doses to the lungs and heart are modifiable and radiation optimization could possibly mitigate postoperative complications.

## 2 Materials and methods

### 2.1 Study design

This single center cohort study was based on a database of patients who underwent surgery for esophageal or gastroesophageal junction cancer at Karolinska University Hospital in Stockholm, between October 1, 2008, and March 31, 2020. The last date of follow-up was August 31, 2021.

### 2.2 Study population

**Fig. 1:**
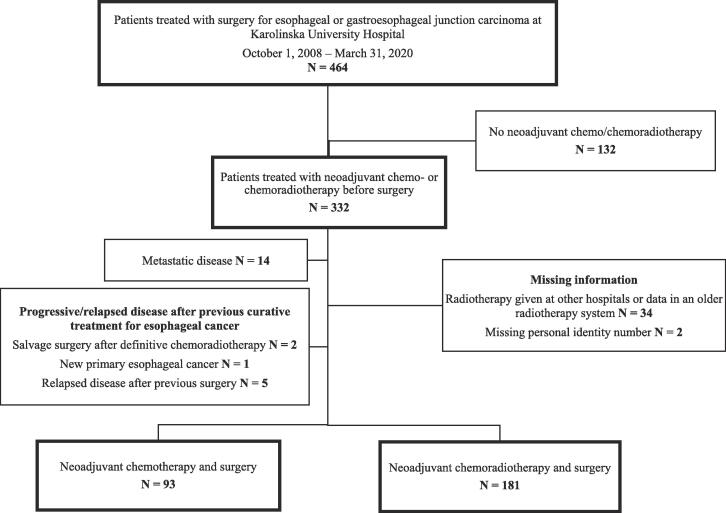
Study selection flow diagram.

All patients treated with neoadjuvant chemo- or chemoradiotherapy and surgery for esophageal or gastroesophageal junction carcinoma, classified as Siewert types I or II [17], were included in the study. For the study selection flow diagram see Fig. 1.

During the study period there were three different prescribed doses to the planning target volume: 41.4 Gy in 23 fractions (95 patients), 40 Gy in 20 fractions (81 patients) and 50 Gy in 25 fractions (5 patients). Macroscopic disease was delineated as gross tumor volume, with the help of a diagnostic FDG-PET/CT scan if available. Para-esophageal nodes next to the primary tumor were included in the target, but no other elective nodal stations. The radiotherapy treatment-plan was done on a computed tomography scan in treatment position and all treatment-plans were prepared in Eclipse (Varian, USA, version 8.0-16.1) using the AAA dose calculation algorithm. The radiotherapy technique used was either three-dimensional conformal radiotherapy (3D-CRT) or volumetric modulated arc radiotherapy (VMAT). The radiotherapy was delivered with 6, 15 or 18MV energy.

### 2.3 Data collection

All data, except details on radiotherapy, were collected from the database of patients treated with surgery for esophageal cancer at our institution. Since data was collected retrospectively the need for informed consent was waived. Any missing values were completed by screening the patients’ electronic medical records. Data on given radiotherapy was collected from the electronic radiotherapy planning system Eclipse. The automatic delineation tool was used to define the lungs. All hearts were delineated according to the atlas by Feng et al. [18] Patient anonymity in the published data has been secured.

No patients were lost to follow-up and the data was complete.

### 2.4 Ethical approval

The Ethical Review Board in Stockholm has approved the study (Diary Number 2018/970-31 and 2020-01459).

### 2.5 Study outcomes

The primary outcome was 30-day postoperative complications graded according to the Clavien-Dindo classification [19]. Postoperative complications were compared between patients treated with nCRT and nCT. The relation of postoperative complications to radiation doses to the lungs and heart was analyzed. The difference in postoperative outcomes between nCRT and nCT was further analyzed in the secondary outcomes: duration of in-hospital care, need for intensive care, and postoperative 30-day, 90-day and 1-year mortality. Survival time was defined as the time from surgery to date of death or last date of follow-up, whichever came first.

### 2.6 Statistical analyses

Stata (version 16.0) and Matlab (version R2018b) were used for the analyses. Differences between treatment groups were analyzed with *χ*^2^ or Fisher’s exact test for categorical variables and the Wilcoxon Rank Sum test for continuous variables. Survival time was estimated with the Kaplan-Meier method and the log-rank test used to assess differences between the two groups.

Postoperative complications within 30 days were analyzed with univariable and multivariable logistic regression. Complications analyzed were those that needed medical treatment or intervention, i.e., Clavien-Dindo ≥ 2 and severe postoperative complications, i.e., Clavien-Dindo ≥ 3b, as well as pulmonary complications, defined according to Low et al. [20], anastomotic leak, and postoperative atrial fibrillation. In the development of a multivariable model, covariates were selected based on possible causal relationships through a directed acyclic graph analysis. Covariates included in the multivariable model were neoadjuvant type (neoadjuvant chemotherapy vs chemoradiotherapy), surgical approach, type of surgery, chemotherapy regimen, ASA score, age, and clinical T- and N-stage. The logistic regression model was assessed with the Pearson’s Goodness-of-Fit test and considered acceptable if *p* > 0.05. The results from the analyses were regarded as statistically significant if the *p* value was <0.05.

For the group of patients treated with nCRT and surgery, univariable and multivariable logistic regression was performed separately, and the radiotherapy dose distribution considered as a predictor of severe postoperative complications, pulmonary complications, and atrial fibrillation. The doses to the lungs and heart were extracted from the treatment planning system as dose-volume histograms (DVH). The DVHs were represented in the logistic regression models by a few alternative summary measures, namely the equivalent uniform dose (EUD) [21], damaged volume (DV) from the critical volume model [22] (see **6. Appendix Eq. (1)** and **Eq. (2)** for the equations for EUD and DV, respectively), and the parameters commonly used as planning aims in radiotherapy treatment planning, i.e., the mean lung dose (MLD), lung V_20Gy_, mean heart dose, heart V_30Gy_ and heart V_40Gy_. All summary measures of the dose distribution were tested based on both relative and absolute volume. Given the smaller cohort of patients, the number of variables included in the multivariable model was constrained to three and was only performed if a significant DVH summary measure could be found in the univariable analysis. The dose distribution summary measure of greatest significance for each endpoint in the univariable analysis, ASA classification and type of surgery were selected for the multivariable analysis.

## 3 Results

### 3.1 Baseline demographics and treatment

During the study period, 181 patients (66 %) received nCRT and 93 patients (34%) nCT followed by surgery for esophageal or gastroesophageal junction cancer. The two groups did not differ regarding baseline characteristics and clinical tumor- and node-stage distributions; however, distal location of the tumor and adenocarcinomas were more common in the nCT-group. With regards to treatment approach, minimally invasive surgery was more common in the nCRT-group, platinum/taxane regimens were primarily used in the nCRT-group and 5-fluorouracil(5-FU)/leucovorin-/oxaliplatin/docetaxel regimen (FLOT-regimen), and epirubicin/platinum/capecitabine regimen (MAGIC-regimen) were only used in the nCT-group (Table 1).

**Table 1:** Baseline patient and tumor characteristics and treatment distribution of patients treated with neoadjuvant chemo- or chemoradiotherapy and surgery for esophageal cancer.

	**All patients**	**Neoadjuvant chemotherapy and surgery Number (%)**	**Neoadjuvant chemoradiotherapy and surgery Number (%)**	***p*-value**
	**Number (%)**			
**All patients**	274	93 (33.9)	181 (66.1)	
**Age, median (IQR)**	64 (59–71)	63 (57–69)	65 (59–71)	0.249
**Sex**				0.248
**Male**	228 (83.2)	74 (79.6)	154 (85.1)	
**Female**	46 (16.8)	19 (20.4)	27 (14.9)	
**ASA classification**				0.065
**1**	96 (35.0)	26 (28.0)	70 (38.7)	
**2**	150 (54.7)	60 (64.5)	90 (49.7)	
**3**	28 (10.2)	7 (7.5)	21 (11.6)	
**BMI, median**	25.1	25.5	25	0.296
**Tumor location**				**0.002**
**Proximal esophagus**	4 (1.5)	1 (1.1)	3 (1.7)	
**Middle esophagus**	37 (13.5)	4 (4.3)	33 (18.2)	
**Distal esophagus**	233 (85.0)	88 (94.6)	145 (80.1)	
**Tumor histology**				**<0.001**
**Adenocarcinoma**	220 (80.3)	83 (89.3)	137 (75.7)	
**Squamous cell carcinoma**	50 (18.3)	7 (7.5)	43 (23.8)	
**Adenosquamous cell carcinoma**	2 (0.7)	2 (2.2)	0 (0.0)	
**Neuroendocrine carcinoma**	2 (0.7)	1 (1.1)	1 (0.6)	
**Clinical T-stage**				0.302
**T-stage 1**–**2**	38 (13.9)	17 (18.3)	21 (11.6)	
**T-stage 3**	177 (64.6)	58 (62.4)	119 (65.8)	
**T-stage 4**	59 (21.5)	18 (19.4)	41 (22.7)	
**Clinical N-stage**				0.255
**N-stage 0**	95 (34.7)	28 (30.1)	67 (37.0)	
**N-stage +**	179 (65.3)	65 (69.9)	114 (63.0)	
**Surgical approach**				**<0.001**
**Open surgery**	81 (29.6)	44 (47.3)	37 (20.4)	
**Any type of minimally invasive surgery**	193 (70.4)	49 (52.7)	144 (79.6)	
**Type of surgery**				0.367
**Ivor-Lewis**	185 (67.5)	65 (69.9)	120 (66.3)	
**McKeown**	81 (29.6)	24 (25.8)	57 (31.5)	
**Transhiatal**	7 (2.6)	3 (3.2)	4 (2.2)	
**Other**	1 (0.4)	1 (1.1)	0 (0.0)	
**Chemotherapy regimen**				**<0.001**
**5-FU/platinum**	134 (48.9)	50 (53.8)	84 (46.4)	
**Platinum/taxane**	97 (35.4)	2 (2.2)	95 (52.5)	
**5FU/leucovorin/oxaliplatin/docetaxel (FLOT regimen)**	30 (11.0)	30 (32.3)	0 (0.0)	
**Epirubicin/platinum/capecitabine (MAGIC regimen) and other combinations**	13 (4.7)	11 (11.8)	2 (1.1)	

IQR = interquartile range.

BMI = Body mass index.

5-FU = 5-fluorouracil.

### 3.2 Postoperative complications

#### 3.2.1 Neoadjuvant chemoradiotherapy compared to chemotherapy

In univariable and multivariable analyses, the addition of radiotherapy to nCT was neither associated with postoperative complications with a Clavien-Dindo score of ≥2 nor with Clavien-Dindo score of ≥3b (univariable analyses in Supplementary Table S1 and multivariable analyses in Table 2).

**Table 2:** Multivariable analyses of postoperative complications, Clavien-Dindo ≥ 2 and ≥ 3b, after neoadjuvant chemo- or chemoradiotherapy and surgery for esophageal cancer.

	**All patients**	**Postoperative complications,** **Clavien-Dindo ≥ 2**			**Severe postoperative complications,** **Clavien-Dindo ≥ 3b**		
	**Number**	**Number (%)**	**OR**†**(95 %CI)**	***p*-value**	**Number (%)**	**OR**†**(95% CI)**	***p*-value**
**Neoadjuvant oncological treatment**							
**Neoadjuvant chemotherapy**	93	64 (68.8)	1.00		29 (31.2)	1.00	
**Neoadjuvant chemoradiotherapy**	181	120 (66.3)	1.10 (0.51–2.39)	0.812	48 (26.5)	1.03 (0.45–2.38)	0.944

**Surgical approach**							
**Open surgery**	81	57 (70.4)	1.00		29 (35.8)	1.00	
**Any type of minimally invasive surgery**	193	127 (65.8)	**0.23 (0.10**–**0.54)**	**0.001**	48 (24.9)	**0.21 (0.08**–**0.56)**	**0.002**

**Type of surgery**							
**Ivor Lewis**	185	112 (60.5)	1.00		42 (22.7)	1.00	
**McKeown**	81	65 (80.3)	**4.38 (2.10**–**9.21)**	**<0.001**	31 (38.3)	**3.46 (1.73**–**6.92)**	**<0.001**
**Transhiatal**	7	6 (85.7)	2.53 (0.28–23.09)	0.410	4 (57.1)	2.17 (0.42–11.33)	0.359
**Other**	1	1 (100)	–	–	0 (0.0)		

**Chemotherapy regimen**							
**5-FU/platinum**	134	80 (59.7)	1.00		34 (25.4)	1.00	
**Platinum/taxane**	97	72 (74.2)	**3.82 (1.76**–**8.29)**	**0.001**	29 (29.9)	**3.09 (1.26**–**7.57)**	**0.013**
**5-FU/leucovorin/oxaliplatin/docetaxel (FLOT)**	30	24 (80.0)	**6.64 (1.86**–**23.69)**	**0.004**	9 (30.0)	**3.91 (1.07**–**14.30)**	**0.039**
**Epirubicin/platinum/capecitabine (MAGIC) and other combinations**	13	8 (61.5)	1.56 (0.41–5.88)	0.512	5 (38.5)	2.00 (0.50–7.94)	0.326

**ASA score**							
**ASA 1**	96	69 (71.8)	1.00		21 (21.9)	1.00	
**ASA 2**	150	91 (60.7)	**0.51 (0.27**–**0.95)**	**0.034**	40 (26.7)	1.35 (0.69–2.63)	0.377
**ASA 3**	28	24 (85.7)	2.09 (0.60–7.33)	0.248	16 (57.1)	**5.39 (1.97**–**14.71)**	**0.001**
**Age (odds per year)**	274	184 (67.2)	1.03 (1.00–1.07)	0.061	77 (28.1)	0.99 (0.96–1.03)	0.593

**Clinical T-stage**							
**T-stage 1**–**2**	38	27 (71.1)	1.00		15 (39.5)	1.00	
**T-stage 3**	177	118 (66.7)	0.78 (0.33–1.86)	0.582	50 (28.3)	0.63 (0.27–1.47)	0.287
**T-stage 4**	59	39 (66.1)	0.52 (0.18–1.49)	0.222	12 (20.3)	**0.31 (0.11**–**0.92)**	**0.034**

**Clinical N-stage**							
**N-stage 0**	95	61 (64.2)	1.00		26 (27.4)	1.00	
**N-stage +**	179	123 (68.7)	0.96 (0.52–1.77)	0.895	51 (28.5)	0.82 (0.42–1.58)	0.554

† Adjusted for neoadjuvant type (neoadjuvant chemotherapy vs chemoradiotherapy), surgical approach, type of surgery, chemotherapy regimen, ASA score, age, clinical T- and N-stage.

Postoperative complications were further classified into pulmonary complications, anastomotic leak, and postoperative atrial fibrillation. Neither pulmonary complications nor incidence of atrial fibrillation differed between the two treatment groups in univariable (Supplementary Table S2) and multivariable analyses (Table 3**)**. Anastomotic leaks were numerically more common after nCRT than nCT (22.1% vs 17.2%) and almost reached statistical significance in the multivariable analysis (OR = 2.82, 95 %CI:1.00–7.98).

**Table 3:** Multivariable analyses of postoperative pulmonary complications, anastomotic leak, and postoperative atrial fibrillation after neoadjuvant chemo- or chemoradiotherapy and surgery for esophageal cancer.

	**All patients**	**Postoperative pulmonary complications**			**Anastomotic leak**			**Postopertive atrial fibrillation**		
	**N(%)**	**N(%)**	**OR**†**(95% CI)**	***p*-value**	**N(%)**	**OR**†**(95% CI)**	***p*-value**	**N(%)**	**OR**†**(95% CI)**	***p*-value**
**Neoadjuvant oncological treatment**										
**Neoadjuvant chemotherapy**	93	30 (32.3)	1.00		16 (17.2)	1.00		10 (10.8)	1.00	
**Neoadjuvant chemoradiotherapy**	181	65 (35.9)	1.23 (0.56–2.74)	0.605	40 (22.1)	2.82 (1.00–7.98)	0.051	21 (11.6)	2.41 (0.76–7.60)	0.133

**Surgical approach**										
**Open surgery**	81	31 (38.3)	1.00		16 (19.8)	1.00		13 (16.1)	1.00	
**Any type of minimally invasive surgery**	193	64 (33.2)	**0.25 (0.11**–**0.60)**	**0.002**	40 (20.7)	0.60 (0.22–1.61)	0.306	18 (9.3)	0.45 (0.15–1.37)	0.160

**Type of surgery**										
**Ivor Lewis**	185	59 (31.9)	1.00		28 (15.1)	1.00		20 (10.8)	1.00	
**McKeown**	81	35 (43.2)	**2.54 (1.33**–**4.82)**	**0.005**	24 (29.6)	**2.64 (1.30**–**5.35)**	**0.007**	11 (13.6)	1.72 (0.70–4.24)	0.238
**Transhiatal**	7	1 (14.3)	0.13 (0.01–1.29)	0.082	4 (57.1)	5.25 (0.97–28.49)	0.054	0 (0.0)		
**Other**	1	0 (0.0)			0 (0)			0 (0.0)		

**Chemotherapy regimen**										
**5-FU/platinum**	134	40 (29.9)	1.00		24 (17.9)	1.00		19 (14.2)	1.00	
**Platinum/taxane**	97	40 (41.2)	**3.32 (1.49**–**7.37)**	**0.003**	21 (21.7)	1.04 (0.43–2.51)	0.926	7 (7.2)	0.40 (0.12–1.34)	0.139
**5-FU/leucovorin/oxaliplatin/docetaxel (FLOT)**	30	10 (33.3)	**3.45 (1.03**–**11.63)**	**0.045**	7 (23.3)	3.60 (0.85–15.31)	0.083	4 (13.3)	2.58 (0.47–14.06)	0.273
**Epirubicin/platinum/capecitabine (MAGIC) and other combinations**	13	5 (38.5)	2.14 (0.56–8.24)	0.269	4 (30.8)	3.50 (0.80–15.32)	0.096	1 (7.7)	0.96 (0.10–8.83)	0.969

**ASA score**										
**ASA 1**	96	32 (33.3)	1.00		12 (12.5)	1.00		12 (12.5)	1.00	
**ASA 2**	150	46 (30.7)	0.88 (0.49–1.60)	0.682	32 (21.3)	1.85 (0.86–3.96)	0.116	14 (9.3)	0.65 (0.27–1.59)	0.348
**ASA 3**	28	17 (60.7)	**3.42 (1.28**–**9.14)**	**0.014**	**12 (42.9)**	**4.86 (1.70**–**13.88)**	**0.003**	5 (17.9)	1.52 (0.43–5.38)	0.512
**Age (odds per year)**	274	95 (34.7)	1.01 (0.98–1.05)	0.386	56 (20.4)	1.00 (0.97–1.04)	0.891	31 (11.3)	1.05 (1.00–1.11)	0.062

**Clinical T-stage**										
**T-stage 1**–**2**	38	13 (34.2)	1.00		7 (18.4)	1.00		7 (18.4)	1.00	
**T-stage 3**	177	67 (37.9)	1.42 (0.62–3.28)	0.409	37 (20.9)	1.07 (0.39–2.91)	0.901	19 (10.7)	0.38 (0.13–1.12)	0.080
**T-stage 4**	59	15 (25.4)	0.56 (0.20–1.54)	0.259	12 (20.3)	0.91 (0.28–2.94)	0.880	5 (8.5)	0.41 (0.10–1.62)	0.201

**Clinical N-stage**										
**N-stage 0**	95	34 (35.8)	1.00		18 (19.0)	1.00		8 (8.4)	1.00	
**N-stage +**	179	61 (34.1)	0.60 (0.34–1.10)	0.101	38 (21.2)	1.08 (0.52–2.24)	0.834	23 (12.9)	1.59 (0.59–4.25)	0.357

† Adjusted for neoadjuvant type (neoadjuvant chemotherapy vs chemoradiotherapy), surgical approach, type of surgery, chemotherapy regimen, ASA score, age, clinical T- and N-stage.

Open surgery and the McKeown surgical type, as well as chemotherapy-regimens containing taxanes, were associated with increased postoperative complications with a Clavien-Dindo score of ≥ 2, Clavien-Dindo score of ≥3b (Table 2) and increased postoperative pulmonary complications in multivariable analyses (Table 3**)**.

#### 3.2.2 Dosimetric predictors in the neoadjuvant chemoradiotherapy group

In the group of patients treated with chemoradiotherapy, the median prescribed total radiation dose to the planning target volume was 41.4 Gy. The median of the MLD was 6.2 Gy (interquartile range (IQR):4.8–7.9 Gy) and the median of the mean heart dose 20.3 Gy (IQR:15.8–23.7 Gy). With regards to radiotherapy technique, 153 patients were treated with 3D-CRT and 28 with VMAT. Neither radiation doses to the lungs or the heart were associated with any of the analyzed postoperative complications, nor was radiotherapy technique associated with severe postoperative complications or pulmonary complications.

#### 3.2.3 Dosimetric predictors in the predominant curative treatment group

Further analyses were performed for the group of patients who were treated with the study period’s predominant treatment approach: platinum/taxane given concomitantly with radiotherapy (the CROSS regimen) followed by minimally invasive surgery. As only two patients in this group were operated on with a transhiatal surgery they were omitted from the analyses. The total number of patients in this group was 94, of which 70 were treated with 3D-CRT and 24 VMAT. Radiotherapy technique was not associated with severe postoperative complications or pulmonary complications.

In a univariable analysis **(**Supplementary Table S3**)**, postoperative pulmonary complications had a statistically significant association with lung DV using absolute volume (OR = 1.37 per 0.5 l, 95 %CI: 1.04–1.81), which was not seen in the subsequent multivariable analysis (Table 4). No other DVH summary measures had a statistically significant association with good precision with pulmonary complications. Severe postoperative complications and postoperative atrial fibrillation were not associated with radiation doses to the lungs or heart.

**Table 4:** Multivariable analysis of postoperative pulmonary complication in patients treated with neoadjuvant platinum/taxane given concomitantly with radiotherapy (CROSS regimen) followed by minimally invasive surgery for esophageal cancer.

	**All patients**	**Postoperative pulmonary complications**		
	**Number**	**Number (%)**	**OR**†**(95 %CI)**	***p*-value**
**Damaged Volume, D50 = 6.9 Gy** **(absolute volume)**	94	40 (42.6)	1.23 (0.88–1.71)	0.213
**Type of surgery**				
**Ivor Lewis**	60	19 (31.7)	1.00	
**McKeown**	34	21 (61.8)	2.36 (0.83–6.77)	0.104

**ASA score**				
**ASA 1**	37	14 (37.8)	1.00	
**ASA 2**	43	16 (37.2)	1.03 (0.39–2.73)	0.944
**ASA 3**	14	10 (71.4)	4.12 (1.00–16.98)	**0.047**

† Adjusted for damaged volume, type of surgery and ASA score.

### 3.3 Postoperative care and survival

**Fig. 2:**
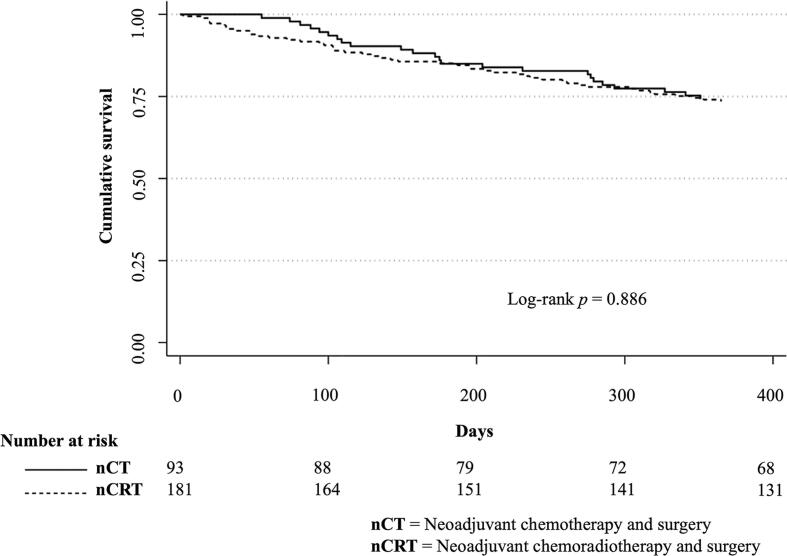
1-year survival after surgery for esophageal cancer.

Neither duration of in-hospital postoperative care nor need for intensive care differed between patients treated with nCRT and nCT (Supplementary Table S4). The median follow-up time was 26.5 months (IQR:11–57 months). The 30-day and 90-day postoperative mortality differed numerically (30-day: 0% in the nCT-group vs 3.3% in the nCRT-group and 90-day: 4.3% in the nCT-group and 8.3% in the nCRT-group), but the differences were not statistically significant (*p* = 0.099 and *p* = 0.316, respectively). One-year survival was 74% in both groups (Fig. 2**)**.

## 4 Discussion

In this study, based on real-world data from an institutional database of patients treated for esophageal cancer, the addition of radiotherapy to neoadjuvant chemotherapy did not increase the incidence or severity of postoperative complications. Neither did we find any association between radiotherapy doses to the lungs and heart and postoperative complications. Taxane-based chemotherapy with, or without the addition of radiotherapy, was associated with increased postoperative complications. As previously shown, surgical approach and type of surgery affected the incidence of postoperative complications [23], [24], [25], [26].

In theory, the addition of radiotherapy to neoadjuvant chemotherapy for esophageal cancer could increase the incidence of postoperative complications by means of radiation-induced tissue damage to the organs in its proximity, the lungs, the heart, and the future gastric conduit. However, in four randomized controlled trials [13], [14], [15], [16], comparing nCRT and nCT, the incidence of postoperative complications was not statistically different, though complications were more severe after nCRT in the NeoRes trial [13]. The non-significant difference in these studies could be a result of small sample sizes, as numerically the incidence of postoperative complications in patients treated with nCRT was higher in all four trials [13], [14], [15], [16]. Two *meta*-analyses have reached different conclusions [27], [28]. Zhao et al. [27] found a two-fold increase in the occurrence of postoperative complications related to nCRT compared to nCT while our group [28] found no difference between nCRT and nCT regarding postoperative morbidity or mortality. Thus, despite the theoretical rationale that the addition of radiotherapy to neoadjuvant chemotherapy could increase postoperative morbidity, results from previous studies are inconsistent and our results do not support this assumption.

Postoperative pulmonary complications have previously been associated with lung dosimetric indices, such as MLD [10], [11], V_5Gy_ [10], [29] and V_10Gy_ [10], [30]_._ We did not find these associations in our cohort as a whole. Compared to previous studies, the median prescribed total radiation dose in our cohort was lower (41.4 Gy vs 44–50.4 Gy) [10], [11], [29], [30] and also the median MLD (6.2 Gy vs 9.6–10.7 Gy) [10], [11]. The generally lower doses in our study could have mitigated the effect of the neoadjuvant radiotherapy. However, in the subgroup of patients treated with the study period́s predominant curative treatment (CROSS-regimen followed by minimally invasive surgery) our study did identify a D50 of 6.9 Gy. This means that the larger the volume exposed to as low doses as 6.9 Gy, the higher the risk of postoperative pulmonary complications. Even though this was not statistically significant in a multivariable analysis after adjusting for ASA score and surgical type, our results and the results of previous studies [10], [29], [30] suggest that when large volumes of the lungs are exposed to low radiotherapy doses in the neoadjuvant setting, this might increase the risk of developing postoperative complications.

To our knowledge, radiation doses to the heart have previously not been shown to be associated with postoperative complications [11], [12] and neither did we find an association. Chemoradiotherapy for esophageal cancer has in earlier studies been shown to impair cardiac function [31], [32]. Hatakenaka et al. [31] demonstrated that high radiation doses (<0.6 Gy vs 3.6 Gy—41.2 Gy) to the left heart ventricle given concurrent with 5-FU/cisplatin was associated with impaired left ventricular function assessed with magnetic resonance imaging. In a previous study by our group [32] nCRT compared to nCT was associated with impaired left ventricular function assessed by echocardiography and plasma NT-proBNP [32]. Nevertheless, it is still unclear whether radiation to the heart, that can cause impaired cardiac function when objectively measured, has an association with clinically relevant early outcomes such as postoperative complications.

Postoperative complications can also be affected by the choice of neoadjuvant chemotherapy regimen used concurrent with radiotherapy. We found taxane-based chemotherapy regimens to be associated with increased postoperative complications, especially pulmonary complications. It has previously been demonstrated in a rat model that paclitaxel can induce alveolar-capillary membrane injury [33]. The combination of carboplatin/paclitaxel has also been associated with a decline in lung function, measured by the diffusing capacity of the lungs for carbon monoxide (DLCO) [34], [35]. Elliott et al. [35] noted a greater decline in the DLCO in patients treated for esophageal cancer with carboplatin/paclitaxel and radiotherapy compared to 5-FU/cisplatin and radiotherapy and the pulmonary physiology changes were associated with increased risk of postoperative pulmonary complications. Furthermore, paclitaxel on its own or together with radiation is a known cause of pneumonitis [36], [37]. In summary, our results and previous clinical and pre-clinical findings lend evidence that taxanes, alone or together with radiotherapy, might be associated with increased risk of postoperative complications by means of worsened pulmonary function.

At our institution, neoadjuvant chemoradiotherapy using a taxane (paclitaxel) and a platinum (carboplatin) became standard treatment for locally advanced esophageal cancer with the CROSS trial [38] and this was our study period’s predominant neoadjuvant treatment. In the CROSS trial, the addition of nCRT to surgery did not increase the incidence of postoperative complications, even though higher postoperative morbidity was noted than previously reported. Postoperative morbidity in our cohort treated according to the CROSS regimen was notably similar to what was reported from the CROSS trial: 43% in our cohort had postoperative pulmonary complications as compared to 46% in the CROSS trial. Likewise, the incidence of anastomotic leak was 21% and 22%, in our cohort and the CROSS trial, respectively [38].

There were some limitations to our study. The long study period and the observational design led to heterogeneity in oncological and surgical treatment in the study cohort. The multivariable analyses included these variables, which in turn minimized possible confounding, though residual confounding cannot be adjusted for. A randomized controlled trial would have had more internal validity; however, we came to the same conclusion as four previous randomized controlled trials comparing nCRT and nCT, and our study has more generalizability because of the real-world setting. In addition, we analyzed dose indices of radiation to the lungs and heart, as these can potentially be optimized. The association of postoperative pulmonary complications with low lung doses in the subgroup of patients treated with the CROSS-regimen merits further analyses in a larger sample size.

## 5 Conclusions

In conclusion, our real-world data supports previous findings that the addition of radiotherapy to neoadjuvant chemotherapy does not increase the incidence or severity of postoperative complications. However, in addition to surgically related variables, taxane-based neoadjuvant chemotherapy regimens were associated with postoperative complications, which raises concern and a need for further research.

## Declaration of Competing Interest

The authors declare that they have no known competing financial interests or personal relationships that could have appeared to influence the work reported in this paper.

## Appendix

Eq. (1).

$\mathrm{EUD}={\left(\sum_{i}v_{i}D_{i}^{a}\right)}^{\frac{1}{a}}$, where *v_i_* is the volume in each bin of the organ dose volume histogram, *D_i_* is the total Dose in each bin, and *a* is a volume effect parameter fitted to the dataset in the logistic regression analysis [21].

Eq. (2).

$\mathrm{DV}=\sum_{i}E\left(D_{i}\right)v_{i}$, where *v_i_* is the volume in each bin of the organ dose volume histogram, *D_i_* is the total Dose in each bin and $E\left(D_{i}\right)=\frac{D_{i}/D_{L50}-1}{1+{\left(D_{i}/D_{L50}-1\right)}^{2}}+1$ when *D_i_* < 2*D_L50_*, and E(*D_i_*) = 1 when *D_i_* ≥ 2*D_L50_*. Here, *D_L50_* is the dose causing a 50% local effect [22].

## References

[1] Obermannová R., Alsina M., Cervantes A., Leong T., Lordick F., Nilsson M. (2022). Oesophageal cancer: ESMO Clinical Practice Guideline for diagnosis, treatment and follow-up. Ann Oncol.

[2] Dhungel B., Diggs B.S., Hunter J.G., Sheppard B.C., Vetto J.T., Dolan J.P. (2010). Patient and peri-operative predictors of morbidity and mortality after esophagectomy: American College of Surgeons National Surgical Quality Improvement Program (ACS-NSQIP), 2005–2008. J Gastrointest Surg.

[3] Viklund P., Lindblad M., Lu M., Ye W., Johansson J., Lagergren J. (2006). Risk factors for complications after esophageal cancer resection: a prospective population-based study in Sweden. Ann Surg.

[4] Takeuchi H., Miyata H., Gotoh M., Kitagawa Y., Baba H., Kimura W. (2014). A risk model for esophagectomy using data of 5354 patients included in a Japanese nationwide web-based database. Ann Surg.

[5] Bailey S.H., Bull D.A., Harpole D.H., Rentz J.J., Neumayer L.A., Pappas T.N. (2003). Outcomes after esophagectomy: a ten-year prospective cohort. Ann Thorac Surg.

[6] Low D.E., Kuppusamy M.K., Alderson D., Cecconello I., Chang A.C., Darling G. (2019). Benchmarking complications associated with esophagectomy. Ann Surg.

[7] Reynolds J.V., Ravi N., Hollywood D., Kennedy M.J., Rowley S., Ryan A. (2006). Neoadjuvant chemoradiation may increase the risk of respiratory complications and sepsis after transthoracic esophagectomy. J Thorac Cardiovasc Surg.

[8] Avendano C.E., Flume P.A., Silvestri G.A., King L.B., Reed C.E. (2002). Pulmonary complications after esophagectomy. Ann Thorac Surg.

[9] Bosch D.J., Muijs C.T., Mul V.E.M., Beukema J.C., Hospers G.A.P., Burgerhof J.G.M. (2014). Impact of neoadjuvant chemoradiotherapy on postoperative course after curative-intent transthoracic esophagectomy in esophageal cancer patients. Ann Surg Oncol.

[10] Cho W.K., Oh D., Kim H.K., Ahn Y.C., Noh J.M., Shim Y.M. (2019). Dosimetric predictors for postoperative pulmonary complications in esophageal cancer following neoadjuvant chemoradiotherapy and surgery. Radiother Oncol.

[11] Wang J., Wei C., Tucker S.L., Myles B., Palmer M., Hofstetter W.L. (2013). Predictors of postoperative complications after trimodality therapy for esophageal cancer. Int J Radiat Oncol Biol Phys.

[12] Thomas M., Defraene G., Lambrecht M., Deng W., Moons J., Nafteux P. (2019). NTCP model for postoperative complications and one-year mortality after trimodality treatment in oesophageal cancer. Radiother Oncol.

[13] Klevebro F., Johnsen G., Johnson E., Viste A., Myrnäs T., Szabo E. (2015). Morbidity and mortality after surgery for cancer of the oesophagus and gastro-oesophageal junction: a randomized clinical trial of neoadjuvant chemotherapy vs. neoadjuvant chemoradiation. Eur J Surg Oncol (EJSO).

[14] Stahl M., Walz M.K., Stuschke M., Lehmann N., Meyer H.-J., Riera-Knorrenschild J. (2009). Phase III comparison of preoperative chemotherapy compared with chemoradiotherapy in patients with locally advanced adenocarcinoma of the esophagogastric junction. J Clin Oncol.

[15] Burmeister B.H., Thomas J.M., Burmeister E.A., Walpole E.T., Harvey J.A., Thomson D.B. (2011). Is concurrent radiation therapy required in patients receiving preoperative chemotherapy for adenocarcinoma of the oesophagus? A randomised phase II trial. Eur J Cancer.

[16] Wang H., Tang H., Fang Y., Tan L., Yin J., Shen Y. (2021). Morbidity and mortality of patients who underwent minimally invasive esophagectomy after neoadjuvant chemoradiotherapy vs neoadjuvant chemotherapy for locally advanced Esophageal squamous cell carcinoma: a randomized clinical trial. JAMA Surg.

[17] Siewert J.R., Stein H.J. (1998). Classification of adenocarcinoma of the oesophagogastric junction. Br J Surg.

[18] Feng M., Moran J.M., Koelling T., Chughtai A., Chan J.L., Freedman L. (2011). Development and validation of a heart atlas to study cardiac exposure to radiation following treatment for breast cancer. Int J Radiat Oncol Biol Phys.

[19] Dindo D., Demartines N., Clavien P.A. (2004). Classification of surgical complications: a new proposal with evaluation in a cohort of 6336 patients and results of a survey. Ann Surg.

[20] Low D.E., Alderson D., Cecconello I., Chang A.C., Darling G.E., D'Journo X.B. (2015). International consensus on standardization of data collection for complications associated with esophagectomy: esophagectomy complications consensus group (ECCG). Ann Surg.

[21] Niemierko A. (1999). A generalized concept of equivalent uniform dose (EUD). Med Phys.

[22] Jin J.-Y., Kong F.-M., Chetty I.J., Ajlouni M., Ryu S., Ten Haken R. (2010). Impact of fraction size on lung radiation toxicity: hypofractionation may be beneficial in dose escalation of radiotherapy for lung cancers. Int J Radiat Oncol Biol Phys.

[23] Mariette C., Markar S.R., Dabakuyo-Yonli T.S., Meunier B., Pezet D., Collet D. (2019). Hybrid minimally invasive esophagectomy for esophageal cancer. N Engl J Med.

[24] Biere S.S., van Berge Henegouwen M.I., Maas K.W., Bonavina L., Rosman C., Garcia J.R. (2012). Minimally invasive versus open oesophagectomy for patients with oesophageal cancer: a multicentre, open-label, randomised controlled trial. Lancet (London, England).

[25] van Workum F., Slaman A.E., van Berge Henegouwen M.I., Gisbertz S.S., Kouwenhoven E.A., van Det M.J. (2020). Propensity score-matched analysis comparing minimally invasive Ivor Lewis versus minimally invasive Mckeown Esophagectomy. Ann Surg.

[26] Brown A.M., Pucci M.J., Berger A.C., Tatarian T., Evans N.R., Rosato E.L. (2018). A standardized comparison of peri-operative complications after minimally invasive esophagectomy: Ivor Lewis versus McKeown. Surg Endosc.

[27] Zhao X., Ren Y., Hu Y., Cui N., Wang X., Cui Y. (2018). Neoadjuvant chemotherapy versus neoadjuvant chemoradiotherapy for cancer of the esophagus or the gastroesophageal junction: a meta-analysis based on clinical trials. PLoS One.

[28] Kumagai K, Rouvelas I, Tsai JA, Mariosa D, Klevebro F, Lindblad M, et al. Meta-analysis of postoperative morbidity and perioperative mortality in patients receiving neoadjuvant chemotherapy or chemoradiotherapy for resectable oesophageal and gastro-oesophageal junctional cancers. Br J Surg 2014;101(4):321-338. 10.1002/bjs.9418. 10.1002/bjs.9418 24493117

[29] Lin J.-B., Hung L.-C., Cheng C.-Y., Chien Y.-A., Lee C.-H., Huang C.-C. (2019). Prognostic significance of lung radiation dose in patients with esophageal cancer treated with neoadjuvant chemoradiotherapy. Radiat Oncol.

[30] Lee H.K., Vaporciyan A.A., Cox J.D., Tucker S.L., Putnam J.B., Ajani J.A. (2003). Postoperative pulmonary complications after preoperative chemoradiation for esophageal carcinoma: correlation with pulmonary dose-volume histogram parameters. Int J Radiat Oncol Biol Phys.

[31] Hatakenaka M., Yonezawa M., Nonoshita T., Nakamura K., Yabuuchi H., Shioyama Y. (2012). Acute cardiac impairment associated with concurrent chemoradiotherapy for esophageal cancer: magnetic resonance evaluation. Int J Radiat Oncol Biol Phys.

[32] Lund M., Alexandersson von Döbeln G., Nilsson M., Winter R., Lundell L., Tsai J.A. (2015). Effects on heart function of neoadjuvant chemotherapy and chemoradiotherapy in patients with cancer in the esophagus or gastroesophageal junction - a prospective cohort pilot study within a randomized clinical trial. Radiat Oncol.

[33] Liu W.J., Zhong Z.J., Cao L.H., Li H.T., Zhang T.H., Lin W.Q. (2015). Paclitaxel-induced lung injury and its amelioration by parecoxib sodium. Sci Rep.

[34] Dimopoulou I., Galani H., Dafni U., Samakovii A., Roussos C., Dimopoulos M.A. (2002). A prospective study of pulmonary function in patients treated with paclitaxel and carboplatin. Cancer.

[35] Elliott J.A., O'Byrne L., Foley G., Murphy C.F., Doyle S.L., King S. (2019). Effect of neoadjuvant chemoradiation on preoperative pulmonary physiology, postoperative respiratory complications and quality of life in patients with oesophageal cancer. Br J Surg.

[36] Bielopolski D., Evron E., Moreh-Rahav O., Landes M., Stemmer S.M., Salamon F. (2017). Paclitaxel-induced pneumonitis in patients with breast cancer: case series and review of the literature. J Chemother (Florence, Italy).

[37] Taghian A.G., Assaad S.I., Niemierko A., Kuter I., Younger J., Schoenthaler R. (2001). Risk of pneumonitis in breast cancer patients treated with radiation therapy and combination chemotherapy with paclitaxel. J Natl Cancer Inst.

[38] van Hagen P., Hulshof M.C.C.M., van Lanschot J.J.B., Steyerberg E.W., Henegouwen M.I.V.B., Wijnhoven B.P.L. (2012). Preoperative chemoradiotherapy for esophageal or junctional cancer. N Engl J Med.

