# Supplementary material for: Postoperative complications after esophagectomy for cancer, neoadjuvant chemoradiotherapy compared to neoadjuvant chemotherapy: A single institutional cohort study
Source: Clin Transl Radiat Oncol. 2023 Mar 4;40:100610. doi: 10.1016/j.ctro.2023.100610 (PMC10018434; doi:10.1016/j.ctro.2023.100610)
Supplement: Supplementary data 1 [file mmc1.pdf]

Table S1 Univariable analyses of postoperative complications, Clavien-Dindo ≥ 2 and ≥ 3b, after neoadjuvant chemo- or chemoradiotherapy and surgery for esophageal cancer.

	All patients	Postoperative complications, Clavien-Dindo ≥ 2			Severe postoperative complications Clavien Dindo ≥3b		
	N(%)	N(%)	OR (95%CI)	*p*-value	N(%)	OR (95% CI)	*p*-value
**Neoadjuvant oncological treatment**							
**Neoadjuvant chemotherapy**	93	64 (68.8)	1.00		29 (31.2)	1.00	
**Neoadjuvant chemoradiotherapy**	181	120 (66.3)	0.89 (0.52-1.52)	0.674	48 (26.5)	0.80 (0.46-1.38)	0.417
**Surgical approach**							
**Open surgery**	81	57 (70.4)	1.00		29 (35.8)	1.00	
**Any type of minimally invasive surgery**	193	127 (65.8)	0.81 (0.46-1.42)	0.463	48 (24.9)	0.59 (0.34-1.04)	0.068
**Type of surgery**							
**Ivor Lewis**	185	112 (60.5)	1.00		42 (22.7)	1.00	
**McKeown**	81	65 (80.3)	**2.65 (1.42-4.93)**	**0.002**	31 (38.3)	**2.11 (1.20-3.71)**	**0.010**
**Transhiatal**	7	6 (85.7)	3.91 (0.46-33.15)	0.211	4 (57.1)	4.54 (0.98-21.09)	0.054
**Other**	1	1 (100)	-	-	0 (0.0)	-	
**Chemotherapy regimen**							
**5-FU/platinum**	134	80 (59.7)	1.0		34 (25.4)	1.00	
**Platinum/taxane**	97	72 (74.2)	**1.94 (1.10-3.44)**	**0.023**	29 (29.9)	1.25 (0.70-2.25)	0.446
**5-FU/leucovorin/oxaliplatin/docetaxel (FLOT)**	30	24 (80.0)	**2.70 (1.03-7.04)**	**0.042**	9 (30.0)	1.26 (0.53-3.02)	0.603
**Epirubicin/platinum/capecitabine (MAGIC) and other combinations**	13	8 (61.5)	1.08 (0.34-3.48)	0.897	5 (38.5)	1.84 (0.56-6.00)	0.313
**ASA score**							
**ASA 1**	96	69 (71.8)	1.00		21 (21.9)	1.00	
**ASA 2**	150	91 (60.7)	0.60 (0.35-1.05)	0.073	40 (26.7)	1.30 (0.71-2.38)	0.397
**ASA 3**	28	24 (85.7)	2.35 (0.74-7.40)	0.145	16 (57.1)	**4.76 (1.95-11.61)**	**0.001**
**Age (odds per year)**	274	184 (67.2)	1.02 (0.99-1.05)	0.128	77 (28.1)	1.00 (0.97-1.03)	0.929
**Clinical T-stage**							
**T-stage 1-2**	38	27 (71.1)	1.00		15 (39.5)	1.00	
**T-stage 3**	177	118 (66.7)	0.81 (0.38-1.76)	0.601	50 (28.3)	0.60 (0.29-1.25)	0.174
**T-stage 4**	59	39 (66.1)	0.79 (0.33-1.92)	0.610	12 (20.3)	**0.39 (0.16-0.97)**	**0.043**
**Clinical N-stage**							
**N-stage 0**	95	61 (64.2)	1.00		26 (27.4)	1.00	
**N-stage +**	179	123 (68.7)	1.22 (0.72-2.07)	0.450	51 (28.5)	1.06 (0.61-1.84)	0.844

Table S2 Univariable analyses of postoperative pulmonary complications, anastomotic leak and postoperative atrial fibrillation after neoadjuvant chemo- or chemoradiotherapy and surgery for esophageal cancer.

	**All patients**	**Postoperative pulmonary complications**			**Anastomotic leak**			**Postoperative atrial fibrillation**		
	**N(%)**	**N(%)**	**OR (95%CI)**	***p*-value**	**N(%)**	**OR (95%CI)**	***p*-value**	**N(%)**	**OR(95%CI)**	***p*-value**
**Neoadjuvant oncological treatment**										
**Neoadjuvant chemotherapy**	93	30 (32.3)	1.00		16 (17.2)	1.00		10 (10.8)	1.00	
**Neoadjuvant chemoradiotherapy**	181	65 (35.9)	1.18 (0.69-2.00)	0.548	40 (22.1)	1.37 (0.72-2.60)	0.343	21 (11.6)	1.09 (0.49-2.42)	0.834
**Surgical approach**										
**Open surgery**	81	31 (38.3)	1.00		16 (19.8)	1.00		13 (16.1)	1.00	
**Any type of minimally invasive surgery**	193	64 (33.2)	0.80 (0.47-1.37)	0.418	40 (20.7)	1.06 (0.56-2.03)	0.855	18 (9.3)	0.54 (0.25-1.16)	0.113
**Type of surgery**										
**Ivor Lewis**	185	59 (31.9)	1.00		28 (15.1)	1.00		20 (10.8)	1.00	
**McKeown**	81	35 (43.2)	1.62 (0.95-2.78)	0.077	**24 (29.6)**	**2.36 (1.27-4.41)**	**0.007**	11 (13.6)	1.30 (0.59-2.85)	0.518
**Transhiatal**	7	1 (14.3)	0.36 (0.04-3.02)	0.344	**4 (57.1)**	**7.48 (1.59-35.2)**	**0.011**	0 (0.0)	-	-
**Other**	1	0 (0.0)	-	-	0 (0)	-	-	0 (0.0)	-	-
**Chemotherapy regimen**										
**5-FU/platinum**	134	40 (29.9)	1.00		24 (17.9)	1.00		19 (14.2)	1.00	
**Platinum/taxane**	97	40 (41.2)	1.65 (0.95-2.85)	0.074	21 (21.7)	1.27 (0.66-2.44)	0.479	7 (7.2)	0.47 (0.19-1.17)	0.104
**5-FU/leucovorin/oxaliplatin/docetaxel (FLOT)**	30	10 (33.3)	1.18 (0.50-2.73)	0.708	7 (23.3)	1.39 (0.54-3.63)	0.494	4 (13.3)	0.93 (0.29-2.97)	0.904
**Epirubicin/platinum/capecitabine (MAGIC) and other combinations**	13	5 (38.5)	1.47 (0.45-4.77)	0.522	4 (30.8)	2.04 (0.58-7.17)	0.268	1 (7.7)	0.50 (0.06-4.10)	0.522
**ASA score**										
**ASA 1**	96	32 (33.3)	1.00		12 (12.5)	1.00		12 (12.5)	1.00	
**ASA 2**	150	46 (30.7)	0.88 (0.51-1.53)	0.661	32 (21.3)	1.90 (0.92-3.90)	0.081	14 (9.3)	0.72 (0.32-1.63)	0.432
**ASA 3**	28	17 (60.7)	**3.09 (1.30-7.37)**	**0.011**	**12 (42.9)**	**5.25 (2.01-13.74)**	**0.001**	5 (17.9)	1.52 (0.49-4.76)	0.471
**Age (odds per year)**	274	95 (34.7)	1.02 (0.99-1.05)	0.229	56 (20.4)	1.02 (0.98-1.05)	0.377	31 (11.3)	1.04 (0.99-1.09)	0.126
**Clinical T-stage**										
**T-stage 1-2**	38	13 (34.2)	1.00		7 (18.4)	1.00		7¨(18.4)	1.00	
**T-stage 3**	177	67 (37.9)	1.17 (0.56-2.44)	0.674	37 (20.9)	1.17 (0.48-2.87)	0.731	19 (10.7)	0.53 (0.21-1.37)	0.193
**T-stage 4**	59	15 (25.4)	0.66 (0.27-1.60)	0.353	12 (20.3)	1.13 (0.40-3.19)	0.816	5 (8.5)	0.41 (0.12-1.40)	0.155
**Clinical N-stage**										
**N-stage 0**	95	34 (35.8)	1.00		18 (19.0)	1.00		8 (8.4)	1.00	
**N-stage +**	179	61 (34.1)	0.93 (0.55-1.56)	0.777	38 (21.2)	1.15 (0.62-2.16)	0.656	23 (12.9)	1.60 (0.69-3.74)	0.274

Table S3 Univariable analyses of postoperative pulmonary complication in patients treated with platinum/taxane given concomitantly with radiotherapy (CROSS regimen) followed by minimally invasive surgery for esophageal cancer.

	**All patients**	**Postoperative pulmonary complications**		
	**N**	**N(%)**	**OR (95%CI)**	***p*-value**
**Sex**				
**Male**	78	32 (41.0)	1.00	
**Female**	16	8 (50.0)	1.43 (0.48-4.29)	0.510
**Type of surgery**				
**Ivor Lewis**	60	19 (31.7)	1.00	
**McKeown**	34	21 (61.8)	**3.49 (1.43-8.50)**	**0.005**
**ASA score**				
**ASA 1**	37	14 (37.8)	1.00	
**ASA 2**	43	16 (37.2)	0.97 (0.39-2.44)	0.954
**ASA 3**	14	10 (71.4)	**4.11 (1.06-15.91)**	**0.038**
**Age (odds per year)**	94	40 (42.6)	1.01 (0.96-1.05)	0.778
**Clinical T-stage**				
**T-stage 1-2**	9	3 (33.3)	1.00	
**T-stage 3**	53	27 (50.9)	2.08 (0.46-9.37)	0.335
**T-stage 4**	32	10 (31.3)	0.91 (0.18-4.48)	0.906
**Clinical N-stage**				
**N-stage 0**	33	13 (39.4)	1.00	
**N-stage +**	61	27 (44.3)	1.22 (0.51-2.93)	0.649
**Damaged volume, D50 = 6.9 Gy (absolute volume)**	94	40 (42.6)	**1.37 (1.04–1.81)**	**0.025**

Table S4 In-hospital care and intensive care after esophagectomy for esophageal cancer.

	**All patients**	**Neoadjuvant chemotherapy and surgery**	**Neoadjuvant chemoradiotherapy and surgery**	***p*-value**
**In-hospital care, median days (IQR)**	15 (10-27)	14 (10-24)	16 (11-27)	0.187
**Intensive care, N (%)**	59 (21.5)	24 (25.8)	35 (19.3)	0.217

IQR = interquartile range
